# Dental College Education

**Published:** 1876-10

**Authors:** E. S. Talbot


### ARTICLE II.

### *Dental College Education.*

BY E. S. TALBOT, CHICAGO.

Perhaps there is no subject that could be brought before this assembly that is entitled to as much time and discussion as the one I have selected for my paper. It is a subject that we are all, or should be, interested in. It is receiving more or less attention at our conventions, both in this country and on the continent. Our dental journals are full of it. And it is our duty, as lovers of our profession, to see that our students, as well as ourselves, keep pace with the strides that are being made to place our banner on the highest pinnacle of fame.

We notice vast improvements in the last twenty-five years, in all branches of our profession. Dental colleges have sprung up, associations have been organized, so that dentists can come together and interchange thoughts and advance new ideas. Practitioners from abroad, and those who live remote from us, can communicate with each other through our dental journals, of which we have an abundace. Not only may we ourselves be thoroughly posted in all the branches of our calling, but we can be always ready and willing to instruct our patients, that they may become enlightened, and thereby aid nature in maintaining her laws of health. Great improvements have been made in our mode of operating, both to the comfort of the patient and operator. Teeth that the dentist would not hesitate to extract, then, are now treated and filled in such a manner as to be of service to the patient for years. That our profession is advancing rapidly, no one will dispute; and the question comes up, Are our institutions of learning capable of instructing the student, that he may grapple with it in its advancing stage, and carry it forward for the next quarter-century ?

To those who are not familiar with the workings of our dental colleges, one would suppose from the amount of controversy that is taking place in our dental journals, and the discussions at our dental conventions for the past ten years, that they were not fit institutions of education—at least one is liable to get that impression; but, gentlemen, such is not the case. They are taught by men of rare attainments, and of the soundest judgement, who have the good of our profession at heart; men whose whole lives have been spent in storing up knowledge, in order to carry out any scheme in regard to dental education that may seem right and good for the benefit of the students, receiving very little sympathy from members of the profession at large, either in the way of encouragement or support by sending students,—these men have stood out boldly and independently in their relations with the profession, and by so doing have sent our representatives all over the world, a credit to our institutions and a benefit to mankind.

From the fact that where there are so many new ideas in regard to the treatment of diseases, and so many new appliances and inventions coming to notice every day, all are tested on their merits, and the good accepted and the bad rejected before they are brought to the notice of the student, that he may not waste time in unnecessary labor, and may rest assured that he will receive nothing but what will be of intrinsic value to him in the future.

There are many dentists whose knowledge in regard to the subjects taught and the manner of illustration is very limited. Many argue that a student will advance faster in a private office than at the college; but, to see how incorrect this statement is, it is only necessary to inquire how many of the practitioners in this country have taken a college course. We have in the neighborhood of thirteen thousand dentists; of this number there are about three thousand who have taken a regular course, and have received a thorough dental education, leaving a balance of ten thousand dentists—men, with a few exceptions, of limited knowledge of those branches in our profession other than mechanical dentistry.

Is it a wonder that we are depreciated by the medical profession, or that many of our patients think that the extraction of teeth is all that is comprehended in the word dentistry? To this class of dentists and those about to enter the profession, I wish to give a clear and concise idea of what our dental colleges consist, and their method of instruction.

Most of our colleges are provided with six professors, who devote themselves to the subjects of chemistry, anatomy, pathology and therapeutics, physiology, operative and mechanical dentistry. Each of these professors lecture every other day, making eighteen lectures a week, together with the clinics that are held at the same college, and also those at the hospitals, which afford most excellent opportunities for students to become familiar with the subjects taught. Not only are they at liberty to attend clinics at the hospital, but when there are operations to be performed on the mouth and jaws at the medical colleges, the students are duly notified, that they may be present.

Our older colleges have a museum connected with them, where have accumulated large assortments of specimens, which for variety and number cannot be excelled. These are acknowledged by men of good authority, not only to facilitate learning, but to be an excellent mode of diagnosing disease.

To those who are in anywise familiar with the study of chemistry, it will plainly appear how utterly impossible it is to comprehend its teachings without the necessary apparatus to illustrate this branch of science; and, as this is becoming a very important branch in connection with dentistry, the student must give the subject a share of his time and attention.

In order that this object may be carried out, the occupant of this chair delivers a thorough and systematic course of lectures, aided by numerous appliances, commencing with the forces that act upon matter and the causes that govern these forces. As the student progresses, the nomenclature is taken up and he is taught the language of chemists, so that he may more easily understand the results that take place by the unity of two or more elements, and the compounds that have been formed by the result of that union.

Anatomy and surgery are generally confined to one chair for the reason that surgery cannot be successfully practiced without a thorough knowledge of the anatomy of the body.

This course of lectures commences with the bones, giving a minute anatomy of all the organs and tissues of the body dwelling more particularly on the head, face and parts connected therewith, and are not only illustrated by preparations, models, drawings, &c., but by dissections, on the cadavers of which they have plenty of material. That the student may most profitably pursue the study of anatomy, and impress the subject on his memory, it is necessary that he should have access to the dissecting room, for which ample accommodations are afforded.

Clinical instruction in the diagnosing of disease, and surgical operations on the mouth and jaws, are performed by this chair on one or two half-days of each week, so that the student may familiarize himself with those specialties. The lecture from the chair on pathology and therapeutics embraces the general knowledge of disease and the art of applying remedies. Although the student does not receive as thorough instruction in diagnosing diseases of the body and treatment as is taught in the medical college, yet he receives sufficient knowledge to enable him to perform such operations as would be likely to come under his notice in every-day practice, thoroughly instructing the student on the causes of inflammation, manner of procedure, and the changes that the tissues undergo until disorganized lymph has formed and pus has worked its way to the surface. Dislocation and fractures of the jaw receive considerable attention from this chair, as well as from the professor of surgery.

This branch of science requires the attention of the student one-half of the course, while the balance of the term is taken up with the classification of remedies and the application of remedies to diseased parts to assist nature and enable her to return to health. The chemical composition of the anæsthetics, chloroform, ether and nitrous oxide; how they are manufactured ; the best modes of administration ; the effect on the patient; the proper remedies to be applied and the means to be used in case the patient is found in a sinking condition,—all are impressed upon the students in such a manner that it would be difficult to forget.

Physiology and microscopic anatomy receive a prominent share of attention. This branch of science which treats of the functions of the body in a healthy state is clearly demonstrated to the student by aid of drawings, models, and a vast amount of preparations that are furnished by the college museum ; and with the aid of the microscope the student is enabled to observe the cell, which is the beginning of all animal and vegetable matter, and even the liquids, a nucleus and nucleolus which are contained in it. We are also enabled to observe the composition of the blood, the white and red corpuscles, the liquor sanguinis, its passage from the heart to every part of the body, the action of osmosis that takes place in the capillaries and its return to the heart, how by this action every part of the body receives its proper nourishment. The functions of each part of the body, and the chemical action that takes place, receive their appropriate amount of attention, so that the student will obtain a general knowledge of how man "lives and moves and has his being."

We now come to the chairs of Operative and Mechanical Dentistry, the two most important branches of our profession. Without mechanical skill and ability to carry out our plans, we are entirely worthless as dentists; therefore, the student who contemplates the practice of dentistry for his calling in life, should have a certain amount of mechanical ingenuity, as well as knowledge in other branches of our profession, to enable him to carry out his work to perfection. From these chairs the student is taught how to arrange his office to the best advantage, how to conduct himself in it, the duty he owes his patients, and the best modes of treating and preserving the natural teeth, and, as a last resort, the extraction and substitution of artificial dentures.

Confining himself strictly to the lectures, and applying the knowledge there obtained with practice in the infirmary, he soon begins to notice vast improvement; he becomes deeper interested every day, and, with encouragement from faculty, he will gain that just reward that is due him.

On matriculating the student's name is placed on the demonstrator's list, and in regular order patients are assigned to him for treatment, and he is expected to place the mouth in a perfect condition, and, after passing inspection by the demonstrator, if found satisfactory the patient is dismissed; if, on the contrary, the work has not been performed in a creditable manner, the patient is returned to the student, the defects pointed out, assistance given if required; and it is hardly necessary to say, the work does not have to be performed a third time.

Does the private office afford such facilities to learn operative dentistry as this? or can a more thorough or systematic course of instruction be obtained elsewhere? The extracting department affords inducements not to be thrown away. The student here finds plenty of opportunity to become quite an expert in this branch of dental science.

We pass now from operative to a few remarks on mechanical dentistry. It is here the student is afforded an opportunity to display his artistic skill as a mechanic. Here he is taught metallurgy in all its branches; he is here informed where the different ores may be obtained, how they are removed from their hidden recesses in the rocks, the process of separating the ore from the debris, and the changes that take place while passing through the smelting process, afterwards the separating and refining and preparation for the market, one of the requirements of graduation being that the student must deposit a case on metal. In the fulfillment of this requirement an active rivalry exists between the students with regard to the best skill displayed in said deposit.

Through the generosity of the faculty, this department is furnished with material free of charge, so that the student can spend the whole of that part of the course devoted to mechanical dentistry in familiarizing himself with the more difficult parts of this branch, which is under the supervision of a demonstrator who is always ready and willing to assist in any part the student may not understand.

But those who have taken one or more courses are soon convinced that the time allotted was not sufficient to digest all that the students are expected to comprehend, to say nothing of the time required for operative and mechanical dentistry. For this reason, the faculty have seen fit to establish a spring and fall course in which the student may devote the entire time to actual practice, with the exception of one hour each day, at which time is delivered a lecture on some subject pertaining to the immediate wants of the students, thus affording facilities superior to the former mode of instruction. We will admit that all who graduate from our dental colleges are not of such material as we would like them to be, but to expect that all are equally qualified is to demand what no college can guarantee. Some will advance faster than others, stand a good examination, and make a poor showing in practice, while others will plod along, appear to be dull, and yet stand at the head of our profession, examples worthy of all imitation. It is for the student to decide whether he will become a shining light or a stumbling-block in the way of those who seek for a higher and nobler aim.

Every practitioner of dentistry will draw to him the class of patients he is qualified to operate upon, and it should be the ambition of every student who anticipates the practice of dentistry for his calling in life, to obtain the highest degree of proficiency that this country affords; and when he has completed his course and has received his reward in the degree of Doctor of Dental Surgery, he may be enabled more confidently to enter the field with a better assurance of success than it would be possible for him to obtain from a private office.—*Transactions of Illinois State Dental Society.*

---

### ARTICLE III.

### *Prevention and Treatment of Approximate Decay.*

